# Efficacy and safety of Mazdutide on weight loss among diabetic and non-diabetic patients: a systematic review and meta-analysis of randomized controlled trials

**DOI:** 10.3389/fendo.2024.1309118

**Published:** 2024-02-14

**Authors:** David Lubasi Nalisa, Nelson Cuboia, Eman Dyab, Idongesit Linus Jackson, Habimana Jean Felix, Pantaleon Shoki, Mary Mubiana, Mariam Oyedeji-Amusa, Luís Azevedo, Hongwei Jiang

**Affiliations:** ^1^ Department of Metabolism and Endocrinology, Endocrine and Metabolic Disease Center, The First Affiliated Hospital, and College of Clinical Medicine of Henan University of Science and Technology, Luoyang, China; ^2^ Adult Hospital Internal Medicine Department, Endocrine Unit, The University Teaching Hospitals, Lusaka, Zambia; ^3^ Center for Health Technology and Service Research (CINTESIS) & Health Research Network Associated Laboratory (RISE), University of Porto, Porto, Portugal; ^4^ Pharmaceutics Department, Faculty of Pharmacy, University of Tripoli, Tripoli, Libya; ^5^ Department of Clinical Pharmacy and Biopharmacy, Faculty of Pharmacy, University of Uyo, Uyo, Akwa Ibom State, Nigeria; ^6^ Directorate of Research and Community Health-Ruli Higher Institute of Health -Saint Rose de Lima (RHIH), Kigali, Rwanda; ^7^ Business Development and Partnership, CLM Consultants Ltd., Dar es Salaam, Tanzania; ^8^ Department of Disease Control, School of Veterinary Medicine, University of Zambia, Lusaka, Zambia; ^9^ Department of Botany and Plant Biotechnology, University of Johannesburg, Johannesburg, South Africa

**Keywords:** dual agonist-receptor, IBI362, diabetes, Mazdutide, obesity, overweight

## Abstract

**Background:**

Overweight and obesity are increasing global public health problems. Mazdutide is a new dual agonist drug that can potentially reduce weight and blood glucose levels simultaneously. However, the synthesis of evidence on the efficacy and safety of this drug is scarce. Therefore, this study aimed to synthesize evidence on the efficacy and safety of Mazdutide compared to placebo on weight reduction among adults with and without diabetes.

**Methods:**

We conducted a systematic review and meta-analysis of randomized controlled trials (RCTs). Data were retrieved from six electronic databases: PubMed, Web of Science, Scopus, Cochrane Library, ClinicalTrial.gov, and Google Scholar, and manually searched from the included references. The data were synthesized using a random effect model. This analysis was performed in the R programming language using the Meta package.

**Results:**

A total of seven RCTs involving 680 participants were included in this study. Mazdutide was more effective in reducing body weight (mean difference [MD]= -6.22%, 95% confidence interval [CI]: -8.02% to -4.41%, I^2^ = 90.0%), systolic blood pressure (MD = -7.57 mmHg, 95% CI: -11.17 to -3.98 mmHg, I^2^ = 46%), diastolic blood pressure (MD = -2.98 mmHg, 95% CI: -5.74 to -0.22 mmHg, I^2^ = 56%), total cholesterol (MD = -16.82%, 95% CI: -24.52 to -9.13%, I^2^ = 61%), triglycerides (MD = -43.29%, 95% CI: -61.57 to -25.01%, I^2^ = 68%), low-density lipoprotein (MD= -17.07%, 95% CI: -25.54 to -8.60%, I^2^ = 53%), and high-density lipoprotein (MD = -7.54%, 95% CI: -11.26 to -3.83%, I^2^ = 0%) than placebo. Mazdutide was associated with reduced hemoglobin A1c (HbA1c) and fasting plasma glucose in participants with type 2 diabetes. In the subgroup and meta-regression analyses, weight reduction was more significant in non-diabetics compared to diabetics, and in those who received a longer treatment duration (24 weeks) than in those on shorter durations (12-20 weeks). Participants who received Mazdutide had a higher risk of transient mild or moderate gastrointestinal side effects.

**Conclusion:**

Mazdutite appears to be effective in weight reduction among patients with and without diabetes, and it has an advantage over other associated comorbidities. However, it was associated with mild or moderate gastrointestinal side effects.

**Systematic review registration:**

https://www.crd.york.ac.uk/prospero/display_record.php?RecordID=403859, identifier CRD42023403859.

## 1 Introduction

Overweight and obesity are global public health problems that have been increasing over the years. According to the World Obesity Atlas, 764 million people in the world were obese in 2020, and this number is expected to increase to one billion by 2030 (1). The estimated worldwide economic impact of obesity is $2.0 trillion, corresponding to 2.8% of the world’s gross domestic product (2). Obesity has a negative impact on life expectancy and quality of life, as it is associated with many health complications (3). Obese individuals are more likely to develop comorbid conditions such as cardiovascular disease, type 2 diabetes, respiratory, gastrointestinal, musculoskeletal, and psychological disorders (3). The treatment of obesity attempts to address and prevent these comorbidities. Obesity is also associated with increased direct and indirect costs, which represent the cost of healthcare services and loss of productivity, respectively (4, 5).

Current conventionally available treatment options for obesity include lifestyle modifications, pharmacotherapy, and surgery (6). Lifestyle modification alone produces modest weight loss, with the possibility of regaining the lost weight in about 12 months (6). Pharmacotherapy is indicated as a complementary intervention when lifestyle measures are ineffective in managing weight and the patient has medical comorbidities (7). In instances where pharmacological treatments also fall short, surgical interventions may be considered for obese patients with a body mass index (BMI) of at least 35 kg/m², particularly those with one or more obesity-related comorbidities and who have not achieved their desired weight loss goals (8).

The ideal treatment intervention for obesity would be one that is safe, effective, convenient, and affordable and can also tackle multiple obesity-associated disorders (9, 10). Various limitations associated with the current available treatment options for obesity have been documented (11). There is a critical need for novel treatments that could potentially overcome these limitations and also address obesity-related comorbidities such as hypertension, type 2 diabetes and hypercholesterolemia.

Mazdutide, also known as IBI362 or LY3305677, is a new dual agonist for the glucagon-like peptide 1 receptor (GLP-1R) and the glucagon receptor (GCGR) (12). It has the potential to simultaneously reduce weight, blood sugar, and other comorbidities associated with obesity (12). Furthermore, due to its dual agonist properties, Mazdutide can counteract the hyperglycemia caused by the GCGR by balancing the activation of GLP-1 and GCGR, hence preserving the desired effects of hemoglobin A1c (HbA1c) and weight loss (13–16). This weight loss effect is expected from the inhibition of satiety and reduced dietary intake caused by GLP-1R agonism and the increase in energy expenditure caused by GCGR stimulation (16).

Different randomized controlled trials (RCTs) have demonstrated the positive effects of Mazdutide on weight loss and the reduction of other comorbidities associated with overweight and obesity among patients with and without diabetes (12, 17, 18). However, to our knowledge, no systematic review has been conducted on the efficacy and safety of Mazdutide for weight reduction among diabetic and non-diabetic patients. Therefore, this study aimed to analyze and synthesize the available evidence on the efficacy and safety of Mazdutide compared to placebo on weight reduction among diabetic and non-diabetic individuals.

## 2 Methods

This systematic review and meta-analysis followed the Preferred Reporting Items for Systematic Reviews and Meta-Analysis (PRISMA) guideline (19).

### 2.1 Eligibility criteria

#### 2.1.1 Inclusion criteria

Based on our Population, Intervention, Comparison, Outcome and Study design (PICOS) criteria, this systematic review included RCTs that evaluated the efficacy or safety of Mazdutide compared to placebo among diabetic and non-diabetic patients with a BMI of at least 20 kg/m^2^ (see **Table 1**).

**Table 1:** Population, Intervention, Comparison and Outcomes (PICOS) Criteria.

P: Population	Adults’ with or without diabetes with BMI ≥ 20kg/m^2^.
I: Intervention	Received Mazdutide treatment, irrespective of dose and treatment duration.
C: Comparison	Placebo
O: Outcomes	Primary outcomes: Change in body weight or incidence of side effects.
	Secondary outcomes: Change in total cholesterol, LDL, HDL, triglycerides, fasting plasma glucose, HbA1c, systolic and diastolic blood pressure.
S: Study design	Randomized Controlled trials

BMI, Body mass index; LDL, low-density lipoprotein; HDL, high-density lipoprotein; HbA1c Hemoglobin A1c.

#### 2.1.2 Exclusion criteria

Studies were excluded if: a) they did not have enough data for analysis; b) they were studies done on non-human subjects; c) they had duplicate data; d) they were letters to the editor.

### 2.2 Search strategy and study selection

We searched six databases for relevant studies in PubMed, Web of Science, Scopus, Cochrane Library, Google Scholar, and ClinicalTrials.gov from inception until December 6, 2023. We had no language restrictions. We also manually searched through the reference list of relevant articles. The complete search query can be found in **Supplementary Table 1**.

The study selection process was performed independently by two reviewers, HJF and MOA. Any discrepancy in the inclusion or exclusion of any study was discussed between the two reviewers to arrive at a consensus. Persistent disagreements were resolved by the research team.

### 2.3 Data extraction

Two reviewers, DLN and ED, independently extracted relevant data from the included studies based on the data extraction form designed for this study. Any differences in the extracted data were discussed between the two reviewers to reach a consensus, and persistent differences were resolved by the research team. The following data were extracted: name of authors, year of publication, title, country, study design, participant recruitment/selection/allocation, sample size, study duration, study setting, patient demographics (such as age, gender, ethnicity, diseases, or conditions), dose of drug administered (Mazdutide), adverse effects, and statistical tests.

### 2.4 Quality assessment of included studies

Two reviewers, DLN and ED, independently assessed the quality of the included studies using the Cochrane Collaboration Tool to determine the risk of bias (ROB2 tool) (20). Persistent disagreements were resolved by the research team. Using the ROB2 tool, five domains were assessed for the risk of bias on the efficacy and safety outcomes, namely: (a) bias arising from the randomization process, (b) bias due to deviations from intended interventions, (c) bias due to missing outcome data, (d) bias in measurement of the outcome, and (e) bias in the selection of the reported outcomes. Based on the results of the ROB2 assessment for each domain, the overall risk of bias for each study was classified as low, some concerns, or high risk of bias for assessed outcomes. We rated the study as having a low risk of bias for intended outcome if it had a low risk of bias in all domains. Some concerns were indicated if there were concerns in at least one domain and a high risk of bias indicated if at least one domain had high risk of bias, or the study had some concerns in multiple domains.

### 2.5 Data analysis

The data were analyzed qualitatively and quantitatively to summarize our findings. The quantitative analysis was conducted using the R programming software version 4.3.1 of June 16, 2023, with the Meta package (21). The pooled effect was estimated using a random-effect model. The measures of effects for efficacy and side effects, respectively, were the mean difference (MD) and the relative risk (RR); each result was accompanied by a 95% confidence interval (CI). The statistical heterogeneity among studies was assessed by the standard chi-squared test, based on the Cochran’s Q statistic, and through the I^2^ statistic (22). We conducted a subgroup, sensitivity, and meta-regression analysis to explore the reasons for heterogeneity and the magnitude of changes in the effect of Mazdutide on weight reduction based on the baseline study variables.

#### 2.5.1 Subgroup analysis

Based on the premise that the effect of Mazdutide on weight reduction can vary depending on different characteristics of the included studies, we stratified studies based on the dose of Mazdutide that was administered (3.0, 4.5, 6.0, 9.0, and 10mg), treatment duration (12, 20 and 24 weeks), stage of RCT (Phase I vs. Phase II), and diabetes status (diabetic vs. non-diabetic). Moreover, we used the standard chi-squared test to examine subgroup differences based on the Cochran’s Q statistic in order to determine whether there were any statistically significant differences in the effect of Mazdutide between the different subgroups. A *p*-value less than 0.05 was considered statistically significant.

#### 2.5.2 Sensitivity analysis and meta-regression

The sensitivity analysis was explored through leave-one-out analysis, and we also performed a meta-regression analysis.

In the leave-one-out analysis, individual studies were systematically removed one at a time from the meta-analysis. The analysis was then repeated each time, leaving out that particular study, to see the impact of removing a study at a time on heterogeneity and the effect size of Mazdutide on weight reduction.

To explore the relationship between the effect of Mazdutide and study characteristics across different studies, we conducted a multivariate meta-regression analysis based on diabetes status (diabetic vs. non-diabetic) and treatment duration (12, 20 and 24 weeks). A *p-*value less than 0.05 was considered statistically significant.

#### 2.5.3 Publication bias

We did not assess the publication bias using funnel plots because the number of included studies was less than ten (23).

The protocol for this systematic review was prospectively registered with PROSPERO (CRD42023403859). Although we adhered to our original registered protocol, modifications to the study population reported here, which now comprised individuals with or without diabetes regardless of BMI, became necessary following recommendations from the peer reviewers.

## 3 Results

### 3.1 Study selection

**Figure 1:**
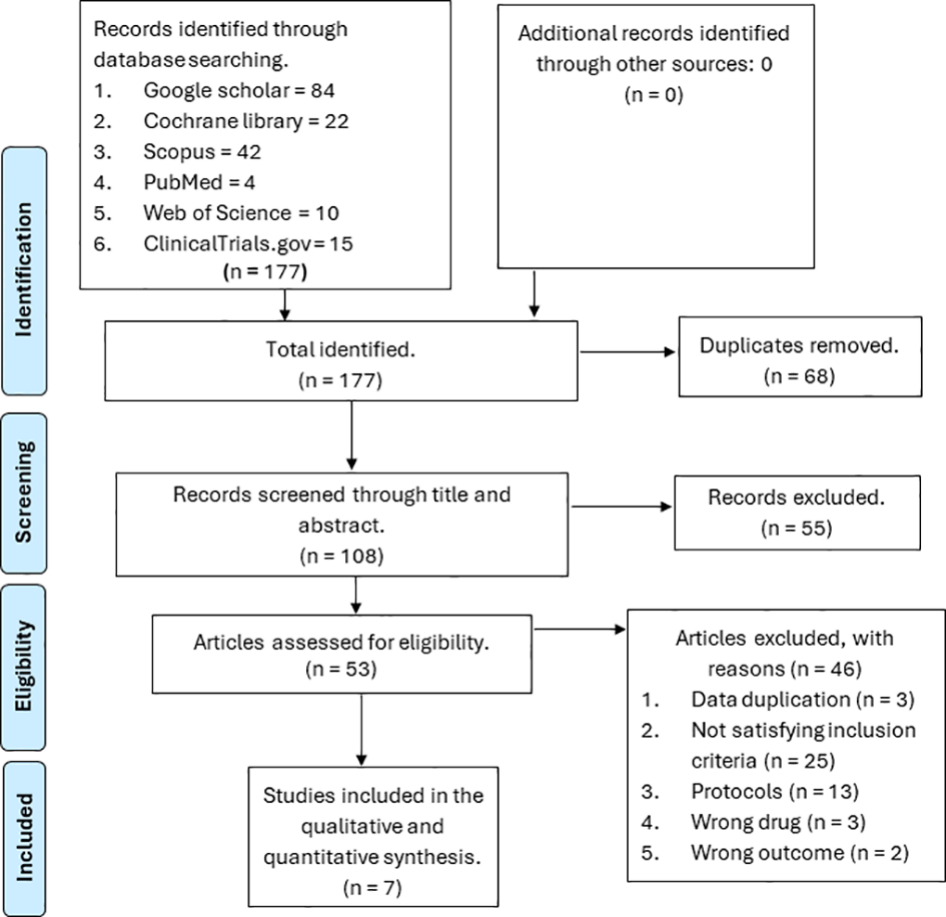
PRISMA flow diagram for the retrieved studies.

We identified a total of 177 references from six databases, namely Web of Science, Scopus, PubMed, ClinicalTrials.gov, Google Scholar, and the Cochrane Library. We removed 68 duplicates; the remaining 108 references were screened through the title and abstract. Out of the 108 screened references, 55 were removed because they were not relevant to our study, and 53 were assessed for eligibility. From the 53 references assessed for eligibility, 46 were excluded based on the following reasons: 25 did not meet our inclusion criteria, 13 were protocols, three had the wrong drugs, two had the wrong outcomes, and three had duplicate data. The remaining seven papers were considered for final inclusion (12, 17, 24–27). The PRISMA flow diagram is displayed in **Figure 1**.

### 3.2 Characteristics of the included studies

Among the seven included studies, five were multicentric (12, 24–27), and two did not provide information on the number of study centers (17). Five were RCTs in phase 1 (12, 17, 24, 27) and two in phase II (25, 26). Five studies were conducted in China (12, 24–27), one in the United Kingdom (17), and one in Germany (17) (see **Table 2**). These studies included a total of 680 participants. Three studies included only participants with type 2 diabetes mellitus (17, 25, 27), and four included non-diabetic subjects (12, 17, 24, 26). All studies compared multiple ascending doses of Mazdutide with placebo. The lowest tested dose was 0.05 mg and the highest was 10mg. In all included studies, Mazdutide was administered once weekly as a subcutaneous injection. The treatment duration ranged from four to 24 weeks. Five studies included patients with a BMI of at least 23 kg/m^2^ (12, 17, 24, 26) and two studies with a BMI of at least 20 kg/m^2^ (25, 27). Among the seven included studies (12, 17, 24–27), five were full-text articles (12, 24–27), while the other two were conference abstracts (17).

**Table 2:** Characteristics of the included studies.

	Aim	Study design	Intervention	Dose(mg)	Comparator	Participants	Sample size	Settings	Country	Treatment duration(weeks)	Registration Number
Ji et al. (24)	To evaluate the safety, tolerability, pharmacokinetics and efficacy of IBI362. up to 6mg	RCT, multiple ascending dose phase 1b	Once-weekly subcutaneous injection of IBI362	3.0, 4.5, and 6.0	Placebo	Adults with overweight (body mass index [BMI] 24 kg/m2) or obesity (BMI 28 kg/m2)	36	6 study centers	China	12	NCT04440345.
Ji et al. (12)	To explore the safety and efficacy of Mazdutide, doses of up to 9 mg and 10 mg.	RCT, multiple ascending dose phase 1b	Once-weekly subcutaneous injection of IBI362	9.0 and 10.0	Placebo	Adults (aged 18-75 years) with overweight (body-mass index [BMI] ≥24 kg/m2) or obesity (BMI ≥28 kg/m2)	24	5 Hospitals	China	12	NCT04440345.
Ji et al. (26)	Evaluate the efficacy and safety of 24-week Mazdutide treatment up to 6 mg in Chinese adults with overweight or obesity.	RCT, double-blind, multiple ascending dose phase 2	Once-weekly subcutaneous injection of IBI362	3.0, 4.5 and 6.0	Placebo	Adults (aged 18-75 years) with overweight (body-mass index [BMI] ≥24 kg/m2) or obesity (BMI ≥28 kg/m2).	248	20 Hospitals	Chine	24	NCT04904913
Jiang et al. (27)	Explore the optimal dosing regimens and assess the safety, tolerability, pharmacokinetics, and efficacy of IBI362 in Chinese patients with T2D.	RCT, multiple ascending dose phase 1b	Once-weekly subcutaneous injection of IBI362	3.0, 4.5, 6.0	Placebo	Adults (aged 18-75years) with T2D and BMI of 20-35kg/m^2^	42	9 studies centers	China	12	NCT04466904.
Zhang et al. (25)	Assessed the efficacy and safety of Mazdutide in Chinese patients with type 2 diabetes.	RCT, double-blind, phase 2	Once-weekly subcutaneous injection of IBI362.	3.0, 4.5, 6.0	Placebo	Adult (aged 18-75years) with T2DM and BMI of ≥20 and ≤40kg/m2	252	32 Hospitals	China	20	NCT04466904
Benson et al. (17)	Evaluate the safety, tolerability, pharmacokinetics, and pharmacodynamics of Mazdutide.	RCT, double-blind, multiple ascending phase 1	Once-weekly subcutaneous injection of LY3305677	0.05, 0.2, 0.5 and 1.5	Placebo	Health subjects, age 20–69 years, BMI 23–40 kg/m2	54	Information not available	Germany	4	NCT03325387
Benson et al. (17)	Evaluate the safety, tolerability, pharmacokinetics, and pharmacodynamics of Mazdutide.	RCT, double-blind, multiple ascending phase 1	Once-weekly subcutaneous injection of LY3305677	0.05mg, 0.2mg, 0.5mg, 1.5mg	Placebo	Adult patients with T2D, age 20–69 years, BMI 25–40 kg/m2	24	Information not available	United Kingdom	12 and 16	NCT03928379

### 3.3 Efficacy of Mazdutide on weight loss

**Figure 2:**
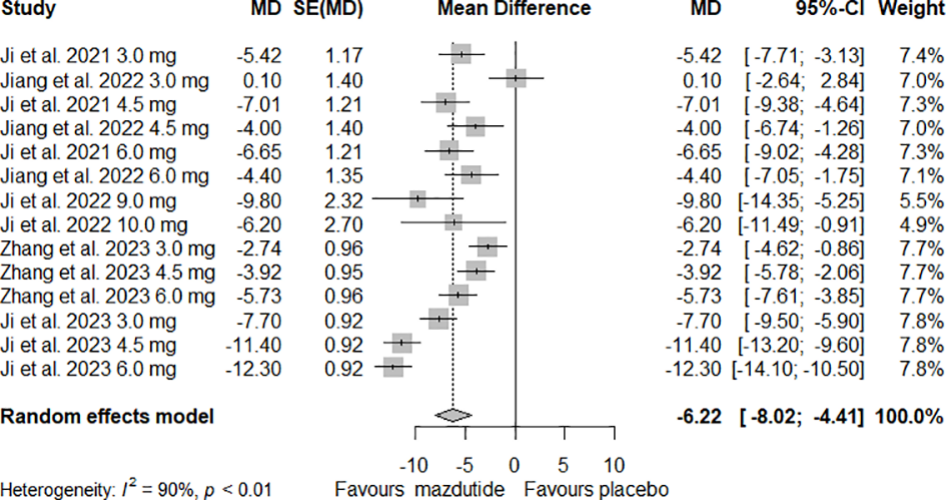
Forest plot of the overall effect of Mazdutide vs placebo on percentage of weight reduction.

For analysis and synthesis of the evidence on the efficacy of Mazdutide compared to placebo on weight loss, five studies with complete data were included (12, 24–27). Our meta-analysis results showed that overall, Mazdutide had a significantly greater reduction in the mean percentage of weight loss than placebo (mean difference [MD]= -6.22%, 95% CI: -8.02% to -4.41%). However, the heterogeneity was very high across included studies, and it was statistically significant (I^2^ = 90.0%, p-value <0.01) (see **Figure 2**).

#### 3.3.1 Subgroup analysis

**Figure 3:**
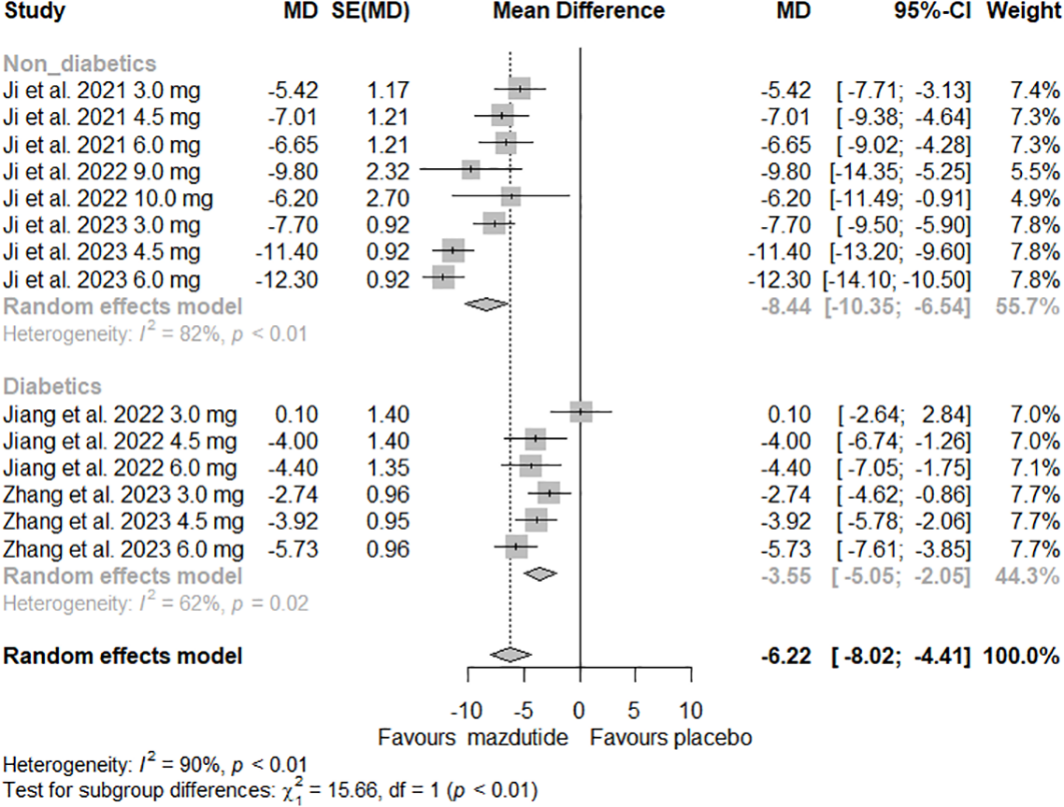
Forest Plot of the effect of Mazdutide vs Placebo on the percentage of weight reduction based on diabetes status of the participants.

**Figure 4:**
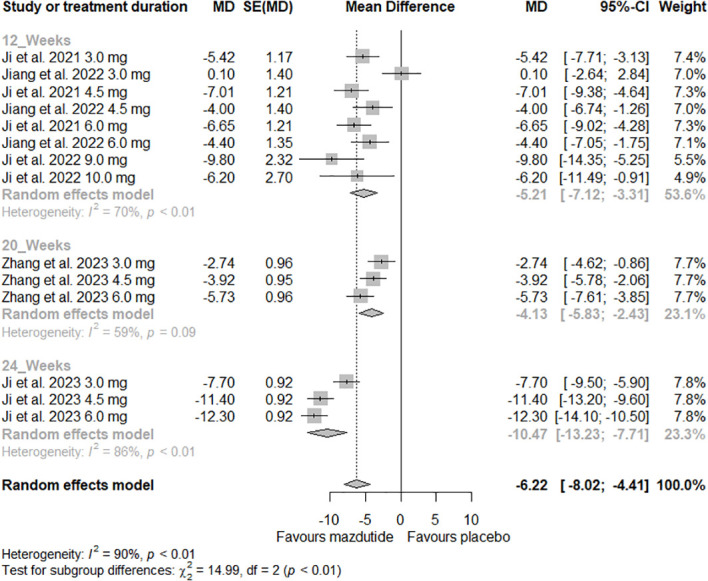
Forest plot of the effect of Mazdutide vs Placebo on the percentage of weight loss based on treatment duration.

In the subgroup analysis, we observed that the effect of Mazdutide on weight loss varied depending on the diabetes status of the participants (see **Figure 3**). Participants without diabetes had greater weight loss (MD=-8.44%, 95% CI -10.35% to -6.54%; I^2^ = 82%) compared to those with diabetes (MD= -3.55%, 95% CI -5.05% to -2.05%; I^2^ = 62%), and this difference was statistically significant (p<0.01). Additionally, we found that the effect of Mazdutide compared to placebo on weight loss varied based on treatment duration. Participants that had a treatment duration of 24 weeks had greater reductions in weight (MD=-10.47%, 95% CI -13.23% to -7.71%, I^2^ = 86%), compared to shorter durations of 12 weeks (MD=-5.21%, 95% CI -7.12% to -3.31%, I^2^ = 70%) and 20 weeks (MD=-4.13%, 95% CI -5.83% to -2.43%, I^2^ = 59%) (see **Figure 4**).

We did not find a statistically significant difference in the effect of Mazdutide on percentage of weight loss based on the administered dose (*p*=0.35) and RCT phase (p=0.26) (see **Supplementary Figures 1**, **2**).

#### 3.3.2 Meta-regression analysis

From the meta-regression analysis, that the effect of Mazdutide was predicted by diabetes status and treatment duration (see **Table 3**). Patients without diabetes had an average weight reduction of -3.99% compared to those with diabetes adjusted for the treatment duration, and this difference was statistically significant (p=0.01). Patients who received Mazdutide for 24 weeks had an average weight reduction of -3.69% compared to those that received treatment for 12 weeks adjusted for diabetes status, and this difference was statistically significant (p=0.01). Patients that received Mazdutide treatment for 20 weeks had an average percentage weight reduction of -1.35% compared to patients that had treatment for 12 weeks adjusted for diabetes status. However, this difference was not statistically significant (*p*=0.40).

**Table 3:** Multivariate meta-regression on the effect of Mazdutide vs Placebo on weight loss.

	Multivariate	
Explanatory variables	MD (95% CI)	*p-value*
Treatment duration		
12 Weeks	Reference	—–
20 Weeks	-1.35(-4.48; 1.79)	0.40
24 Weeks	-3.69(-6.52; -0.86	0.01
Diabetes		
Yes	Reference	—
No	-3.99(-7.05; -0.93)	0.01

MD, Mean Difference; CI, Confidence Interval; Ref, Reference category.

#### 3.3.3 Leave-one-out sensitivity analysis

**Figure 5:**
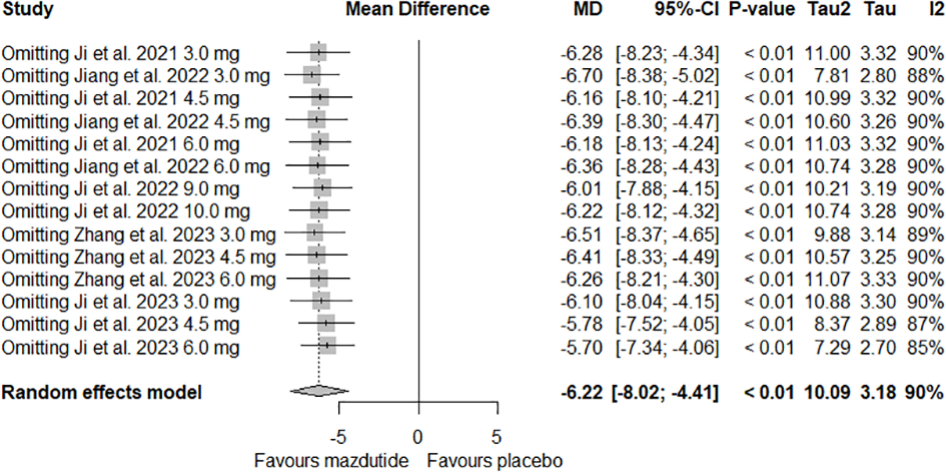
Forest plot of leave-one-out meta-analysis on the effect of Mazdutide vs Placebo on the percentage of weight loss.

In the sensitivity analysis, we found that removing any of the RCTs had no significant effect on heterogeneity and on the percentage of weight loss (see **Figure 5**).

### 3.4 Safety of Mazdutide

Regarding the safety of Mazdutide compared to placebo, seven studies were synthesized for this analysis (12, 17, 24–27). Our meta-analysis showed that patients who received Mazdutide had a higher risk of nausea (risk ratio [RR] =4.22%, 95% CI 2.23% to 7.99%; I^2^ = 0%), vomiting (RR= 4.91%, 95% CI 2.01% to 12.00%, I^2^ = 0%) and decrease in appetite (RR: 2.30%, 95% CI 1.45% to 3.65%, I^2^ = 0%), compared to those that received placebo (see **Supplementary Figure 3**). Moreover, we did not find any statistically significant differences in the occurrence of abdominal distension, diarrhea, hypoglycemia, upper respiratory tract infections, dyspepsia, urinary tract infections, myocardial ischemia, first-degree atrioventricular block, ventricular extrasystoles, supraventricular extrasystoles and sinus tachycardia in patients that received Mazdutide compared to those that received placebo (see **Supplementary Figure 4**). Although, overall, we did not find statistically significant differences in the occurrence of diarrhea among patients who received Mazdutide compared to placebo, in the subgroup analysis, the occurrence of diarrhea was associated with diabetes status. People with diabetes had a higher risk of occurrence of diarrhea (RR=3.84, 95% CI 1.68 to 8.79, I^2^ = 0%) compared to those that did not have diabetes (RR= 0.81, 95% CI 0.28 to 2.40, I^2^ = 64%) (see **Supplementary Figure 3**).

Two cases with serious side effects (myocardial ischemia) were reported among patients who received Mazdutide in a study conducted by Jiang and colleagues (27), and another case (exacerbation of chronic sarcoidosis) was reported by Benson and colleagues (17). However, these cases were considered unrelated to the study drug. Additionally, one participant on the 4.5mg dose of Mazdutide in the Jiang and colleagues’ study discontinued the treatment due to a decrease in appetite; however, this effect was not considered to be related to the medication (27). All remaining cases of side effects were deemed transient and mild to moderate in severity. Five of the included studies reported an increase in heart rate among participants who received Mazdutide (12, 24–27). The magnitude of change from baseline to the end of treatment ranged from 5.0 to 17.4 beats per minute (bpm) on average for participants who received Mazdutide, compared to 1.7 to 4.8 bpm for those who received placebo (12, 24–27). However, there was no increased risk of cardiac events as a result of the elevated heart rates.

### 3.5 Secondary outcomes

Regardless of diabetes status, Mazdutide was associated with reductions in systolic blood pressure (MD= -7.57 mmHg, 95% CI -11.17 mmHg to -3.98 mmHg), diastolic blood pressure (MD=-2.98 mmHg, 95% CI -5.74% to -0.22%, I^2^ = 56%), total cholesterol (MD=-16.82%, 95% CI -24.52% to -9.13%, I^2^ = 61%), low-density lipoprotein (LDL) (MD= -17.07%, 95% CI -25.54% to -8.60%, I^2^ = 53%), triglycerides (MD=-43.29%, 95%CI -61.57% to -25.01%, I^2^ = 68%) and high-density lipoprotein (HDL) (MD=- 7.54%, 95% CI -11.26% to -3.83%, I^2^ = 0%) compared to placebo (see **Supplementary Figure 5**).

Mazdutide was also associated with reductions in fasting plasma glucose (-1.73 mmol/L, 95% CI -2.61 mmol/L to -0.85 mmol/L: I^2^ = 60%) and HbA1c (MD=-1.27%, 95% CI -1.74% to -0.80%, I^2^ = 0%) among people with diabetis.

### 3.6 Assessment of the quality of the included studies

**Figure 6:**
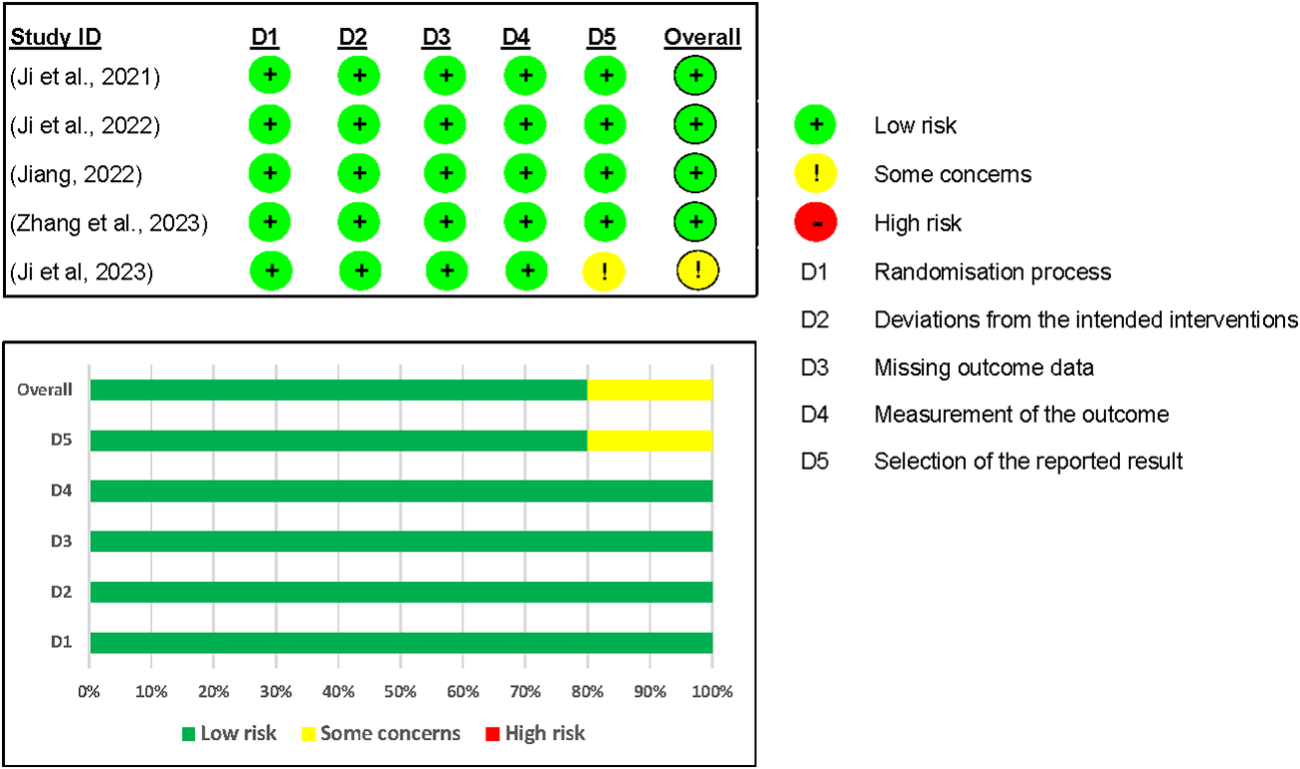
Risk of Bias assessment of Mazdutide vs Placebo on efficacy outcomes.

We included five studies in the assessment of the risk of bias on the efficacy outcomes (12, 24–27). Overall, four out of five studies were graded as having a low risk of bias. However, one study by Ji and colleagues (26) was rated as having some concerns because no information was found in their registered protocol (NCT04904913) on the data analysis plan (see **Figure 6**).

**Figure 7:**
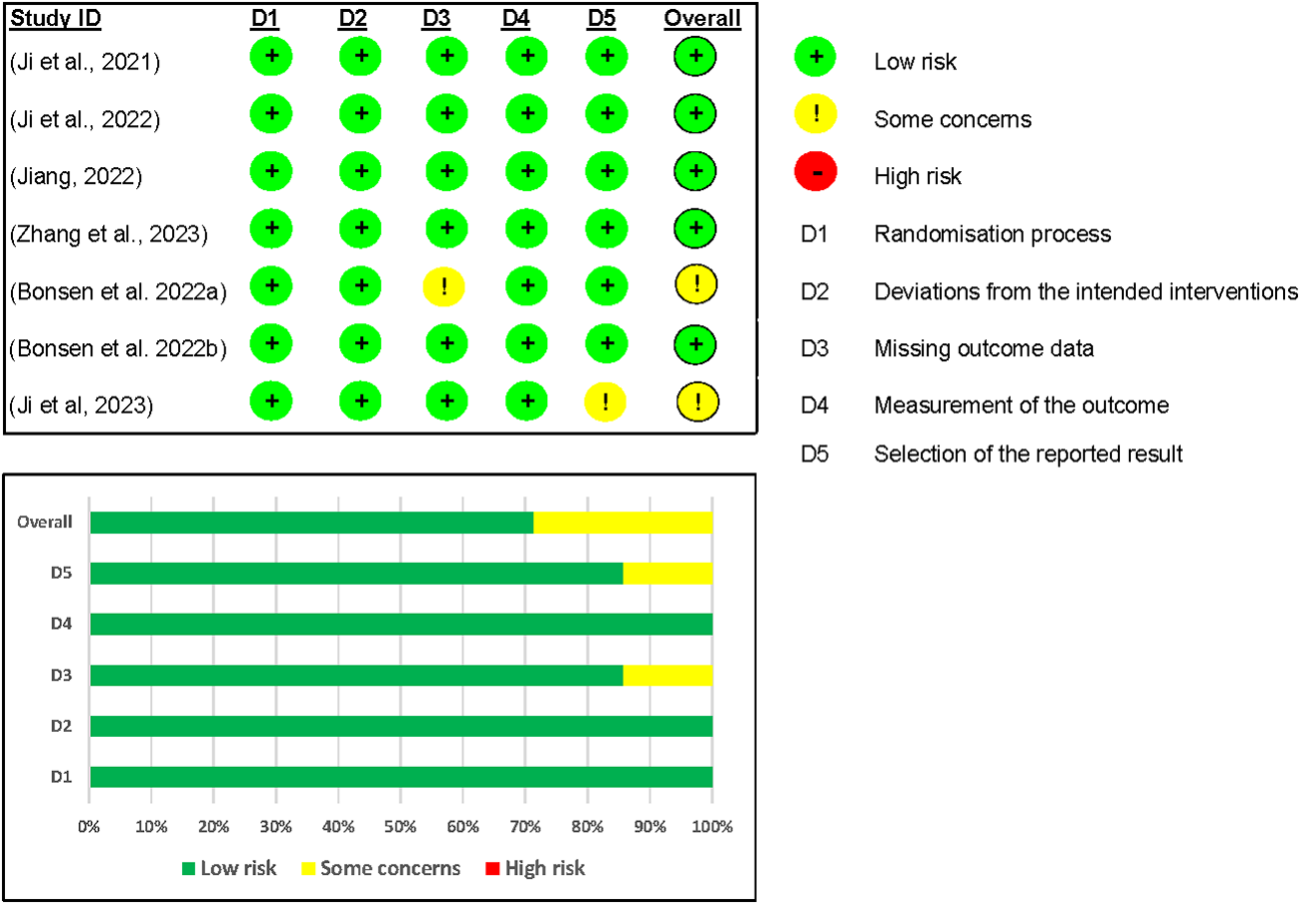
Risk of Bias assessment of Mazdutide vs Placebo on safety outcomes.

In the risk of bias assessment of the safety outcome (12, 17, 24–27), we included seven studies for analysis. Five out of the seven studies were rated as having a low risk of bias overall (12, 17, 24, 25, 27). However, one study by Ji and colleagues (26) was rated as having some concerns because no information was found in their registered protocol (NCT04904913) on the data analysis plan. The Benson and colleagues’ (2022a) study was also rated as having some concerns because outcome data was missing on domain 3 of risk of bias assessment because it was just an abstract (17) (see **Figure 7**).

## 4 Discussion

### 4.1 Key findings

Our study showed that Mazdutide was more effective than placebo in reducing weight, systolic and diastolic blood pressure, total cholesterol, triglycerides, LDL and HDL among patients with and without diabetes. The weight reduction was more significant in non-diabetic participants compared to diabetics. We found that Mazdutide, compared to placebo, was also associated with reductions in fasting plasma glucose and HbA1c among patients with diabetes. Mazdutide was associated with more side effects, primarily gastrointestinal-related disorders, such as decreased appetite, nausea, vomiting and diarrhea. Moreover, all participants on Mazdutide experienced an increase in heart rates. However, these side effects were transient and mild to moderate in severity, and no participant discontinued treatment due to the side effects.

#### 4.1.1 Efficacy

Our results are consistent with previous studies on the efficacy and safety of the GLP-1R agonists and GCGR dual agonists for the treatment of obesity and overweight in individuals with and without diabetes (28–33).

In our study, Mazdutide, compared to placebo, showed an overall significant weight reduction of -6.22% among diabetics and non-diabetics. Interestingly, Mazdutide was also associated with reductions in systolic and diastolic blood pressure, total cholesterol, triglycerides, LDL and HDL, irrespective of diabetes status. In people with diabetes, Mazdutide was associated with reductions in fasting plasma glucose and HbA1c. Notably, weight loss above 5% has been associated with improved glycemic control and other related risk factors (34, 35). Therefore, an initial target weight loss of 5% – 10% of baseline weight within the first six months has been recommended for the management of overweight and obesity in adults (35). These findings suggest that Mazdutide is a promising drug of choice for weight reduction among diabetics and non-diabetics, with or without any comorbidities.

Additionally, we found that the effect of Mazdutide on weight loss was more pronounced in patients without diabetes than those with diabetes. These findings concur with a systematic review and meta-analysis conducted by Vilsbøll and colleagues on the effect of GLP-1R agonists on weight loss among patients with and without diabetes (33). The exact reasons behind the weaker response to GLP-1R agonists in people with diabetes compared to those without diabetes are not fully understood, but there are some possible explanations: a) People with type 2 diabetes have hyperinsulinemia (due to insulin resistance), a condition that can promote fat storage and reduce fat breakdown. b) Concurrent use of anti-diabetic medications known to cause weight gain, such as sulfonylureas and insulin. c) People with diabetes may eat more frequently or in larger quantities due to their inherent fear of hypoglycemia during diabetes treatment; this could result in weight gain or affect their ability to lose weight. d) Reduction of glucosuria in patients on anti-diabetic medication can result in less weight loss and possible weight gain. e) Individuals with type 2 diabetes often have a longer history of obesity, experience changes in their gut bacteria, possess genetic tendencies toward weight gain, and are typically older than those who are obese but not diabetic. Additionally, the effectiveness of GLP-1R agonist treatments for weight loss in people with diabetes may be impacted by their potential difficulties in maintaining exercise routines compared to those without diabetes (36, 37).

Our research indicates that the effect of Mazdutide on weight loss varied based on the treatment duration. The treatment duration of 24 weeks was associated with more significant weight reduction compared to a shorter treatment duration of 12 and 20 weeks. These findings suggest that the duration of treatment with Mazdutide significantly influences its effectiveness in promoting weight loss. Specifically, a longer treatment duration appears to yield better results regarding weight reduction than shorter durations. This implies that for individuals seeking optimal weight loss benefits from Mazdutide, a treatment period of at least 24 weeks may be more effective than shorter durations. It also highlights the importance of considering treatment duration when prescribing Mazdutide for weight management in both diabetic and non-diabetic populations.

In our meta-analysis, we did not find a difference in the weight reduction effect of Mazdutide based on the administered dose. This suggests that Mazdutide might not exhibit a dose-dependent response in diabetic and non-diabetic individuals. However, we observed a slight trend toward a greater effect with higher doses. This possible dose-independent effect of Mazdutide should be evaluated in future studies. Additionally, our findings indicated that the effect of Mazdutide vs. placebo did not vary based on the phase of RCT, suggesting that there was consistency in our findings across different RCT phases.

#### 4.1.2 Safety

Our findings showed that participants who received Mazdutide had more gastrointestinal side effects than those who received placebo. These results correlate with those of a meta-analysis conducted by Guo and colleagues (28) and an RCT by Ambery and colleagues (31). These studies showed that the GLP-1R agonists and glucagon dual agonists were associated with higher risks of occurrence of gastrointestinal side effects compared to placebo (28, 31). The higher occurrence of gastrointestinal side effects might be due to the effect of Mazdutide on multiple gastrointestinal hormone receptors and the central nervous system (38).

The increased heart rate observed in all participants on Mazdutide has been reported in previous studies on the use of mono- or dual- receptor glucagon agonists for weight reduction (31, 39). The increased heart rate with the use of Mazdutide can be explained by the effect of GCGRs on the cardiovascular system (40, 41). We did not find a significant difference in the occurrence of diarrhea between participants without diabetes that received Mazdutite and participants without diabetes that received placebo. This contradicts the findings of previous studies among participants without diabetes, which reported a significantly higher occurrence of diarrhea in those who received GLP-1R agonists compared to the group that received placebo (28, 33). This could be due to our much smaller sample size compared to those of these other studies. However, we found a significant increase in the frequency of diarrhea in participants with diabetes who received Mazdutide compared to those who received placebo.

In our study, we did not find any statistically significant difference in the occurrence of hypoglycemia among participants who received Mazdutide compared to those on placebo. This is in line with the findings of previous studies (28, 32), implying that Mazdutide can effectively reduce weight and achieve glycemic control without increasing the risk of hypoglycemia. This favorable safety profile of Mazdutide, in addition to its once-weekly dosing, offers the advantage of improved treatment adherence.

#### 4.1.3 Limitations and strengths

Our study has several limitations: Considering that the included studies were all from phases I and II RCTs, which are early stages of clinical trials with the primary objective of assessing the safety, best dose, and preliminary efficacy of interventions in small samples of subjects, these results must be interpreted with caution. Additionally, since the data organized by doses on side effects were not available for extraction, we did not assess the effect of different doses of Mazdutide on the occurrence of side effects.

In spite of these limitations, our study has some strengths. To the best of our knowledge, this is the first systematic review and meta-analysis that analyzed and synthesized the evidence on the efficacy and safety of Mazdutide compared to placebo. Furthermore, we included only RCTs, which are considered the gold standard of study design. Lastly, the included studies were multicentric and included diverse populations (diabetics and non-diabetics) who received different doses of Mazdutide; this increased the generalizability of our findings.

## 5 Conclusion

Mazdutide was effective for weight loss in diabetic and non-diabetic patients. Additionally, the drug was effective in reducing blood pressure, total cholesterol, triglycerides, LDL and HDL. Mazdutide was associated with reduced HbA1c and fasting plasma glucose in patients with type 2 diabetes mellitus. However, it was associated with mild or moderate gastrointestinal side effects. With a convenient weekly dose and a favorable safety profile, Mazdutide is a potential drug of choice for weight reduction in people with diabetes (with or without associated comorbidities) and particularly in the non-diabetic population. However, more RCTs from phase III with larger sample sizes are needed to have a definitive answer on the efficacy and safety of Mazdutide on weight reduction among diabetic and non-diabetic individuals.

## Data availability statement

The original contributions presented in the study are included in the article/**Supplementary Material**. Further inquiries can be directed to the corresponding author.

## Author contributions

DN: Conceptualization, Data curation, Investigation, Methodology, Project administration, Resources, Software, Validation, Visualization, Writing – original draft, Writing – review & editing. NC: Data curation, Formal analysis, Methodology, Software, Validation, Visualization, Writing – original draft, Writing – review & editing. ED: Data curation, Investigation, Methodology, Validation, Visualization, Writing – original draft, Writing – review & editing. IJ: Data curation, Formal analysis, Methodology, Software, Validation, Visualization, Writing – original draft, Writing – review & editing. JH: Data curation, Methodology, Resources, Validation, Writing – original draft, Writing – review & editing. PS: Data curation, Methodology, Validation, Writing – original draft. MM: Methodology, Validation, Writing – original draft, Writing – review & editing. MOA: Data curation, Methodology, Validation, Writing – original draft, Writing – review & editing. LA: Methodology, Validation, Writing – review & editing, Formal analysis, Software, Visualization. HJ: Conceptualization, Funding acquisition, Methodology, Supervision, Validation, Writing – review & editing.

## Supplementary material

Supplementary Figure 1 Forest plot of the effect of Mazdutide vs Placebo on the percentage of weight loss based on dose administered.

Supplementary Figure 2 Forest plot of the effect of Mazdutide vs Placebo on the percentage of weight loss based on phase of RCT.

Supplementary Figure 3 Forest plot of gastrointestinal side effects of Mazdutide vs Placebo.

Supplementary Figure 4 Forest plot of the occurrence of non-gastrointestinal side effects of Mazdutide vs Placebo.

Supplementary Figure 5 Forest plot on the effect of Mazdutide vs Placebo on secondary outcomes.
