# Supplementary figures and images for: Efficacy and safety of Mazdutide on weight loss among diabetic and non-diabetic patients: a systematic review and meta-analysis of randomized controlled trials
Source: Front Endocrinol (Lausanne). 2024 Feb 14;15:1309118. doi: 10.3389/fendo.2024.1309118 (PMC10911117; doi:10.3389/fendo.2024.1309118)

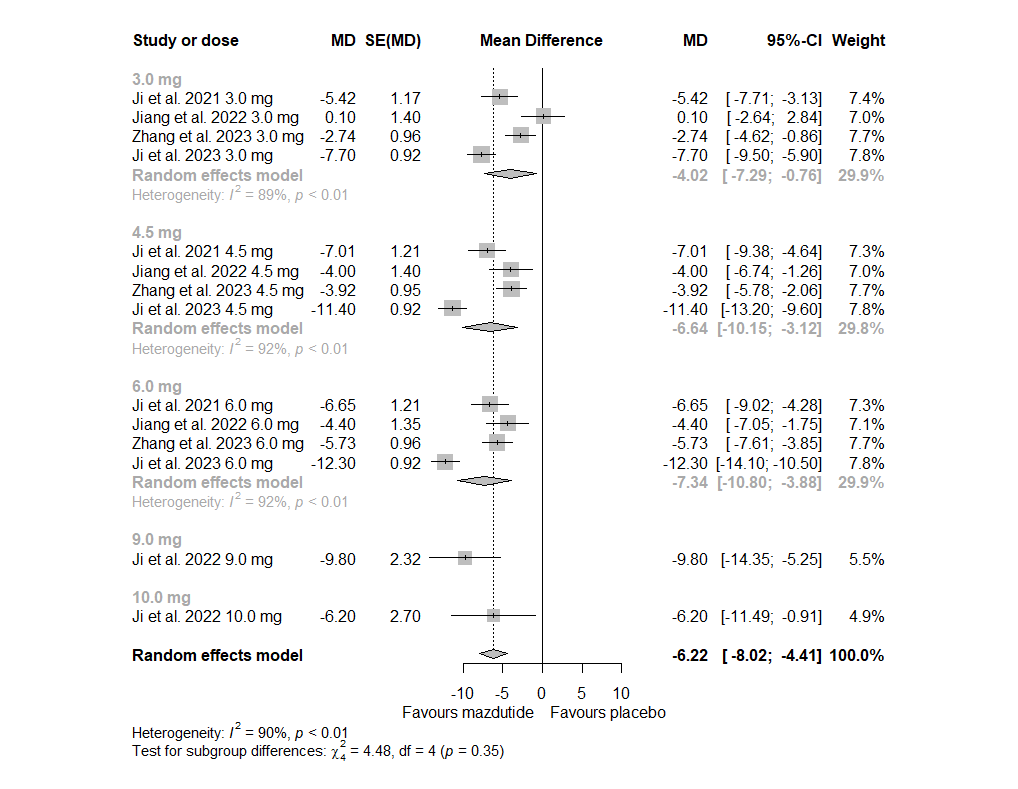

Supplement: Supplementary Figure 1 — Forest plot of the effect of Mazdutide vs Placebo on the percentage of weight loss based on dose administered. [file Image_1.tiff]

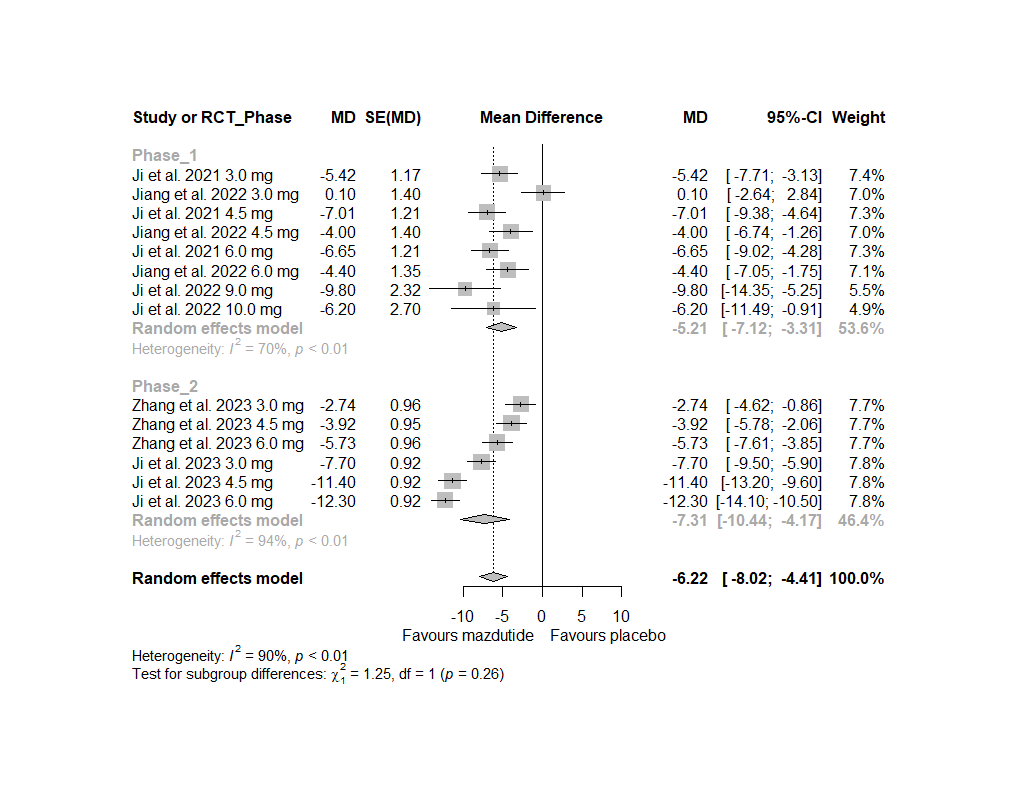

Supplement: Supplementary Figure 2 — Forest plot of the effect of Mazdutide vs Placebo on the percentage of weight loss based on phase of RCT. [file Image_2.tiff]

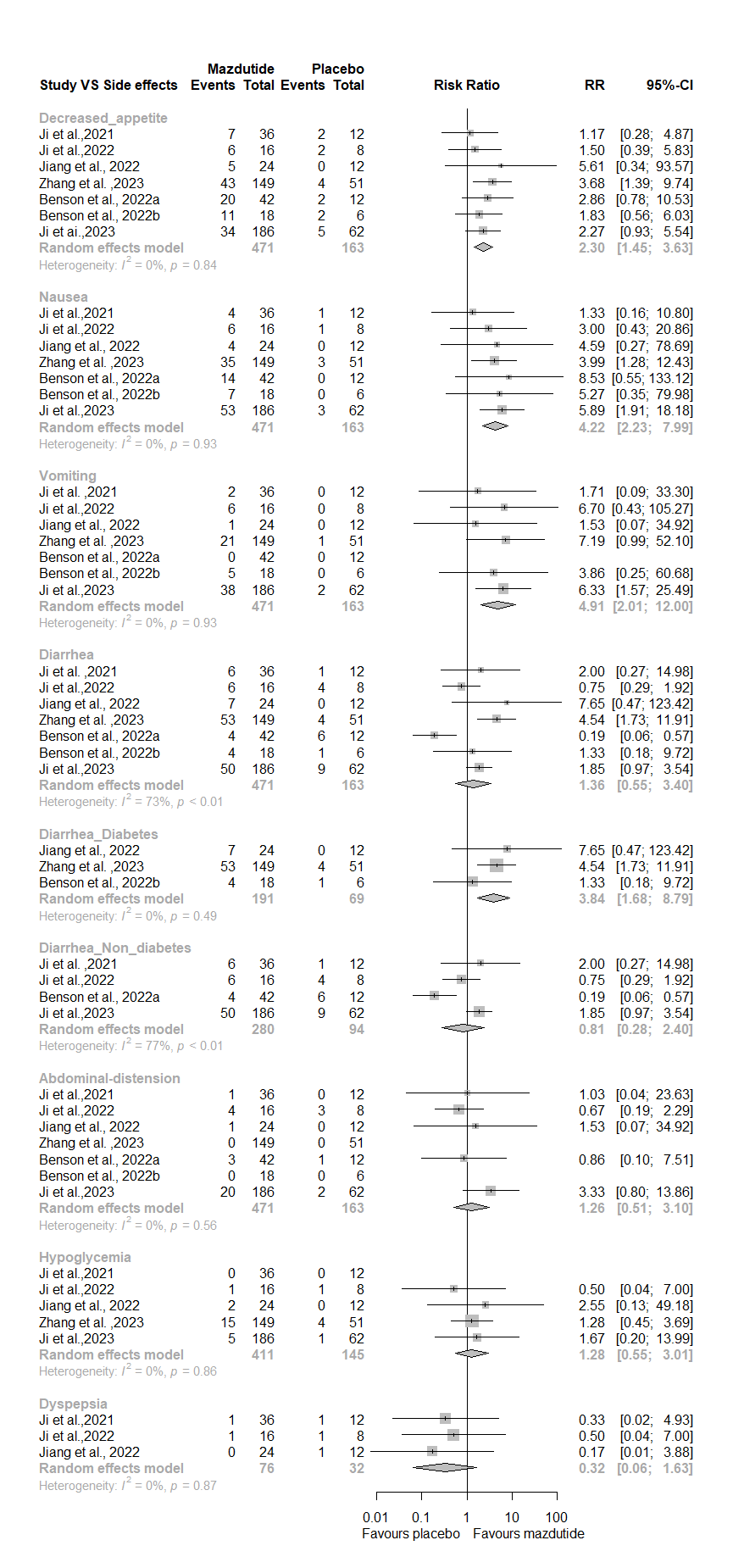

Supplement: Supplementary Figure 3 — Forest plot of gastrointestinal side effects of Mazdutide vs Placebo. [file Image_3.tiff]

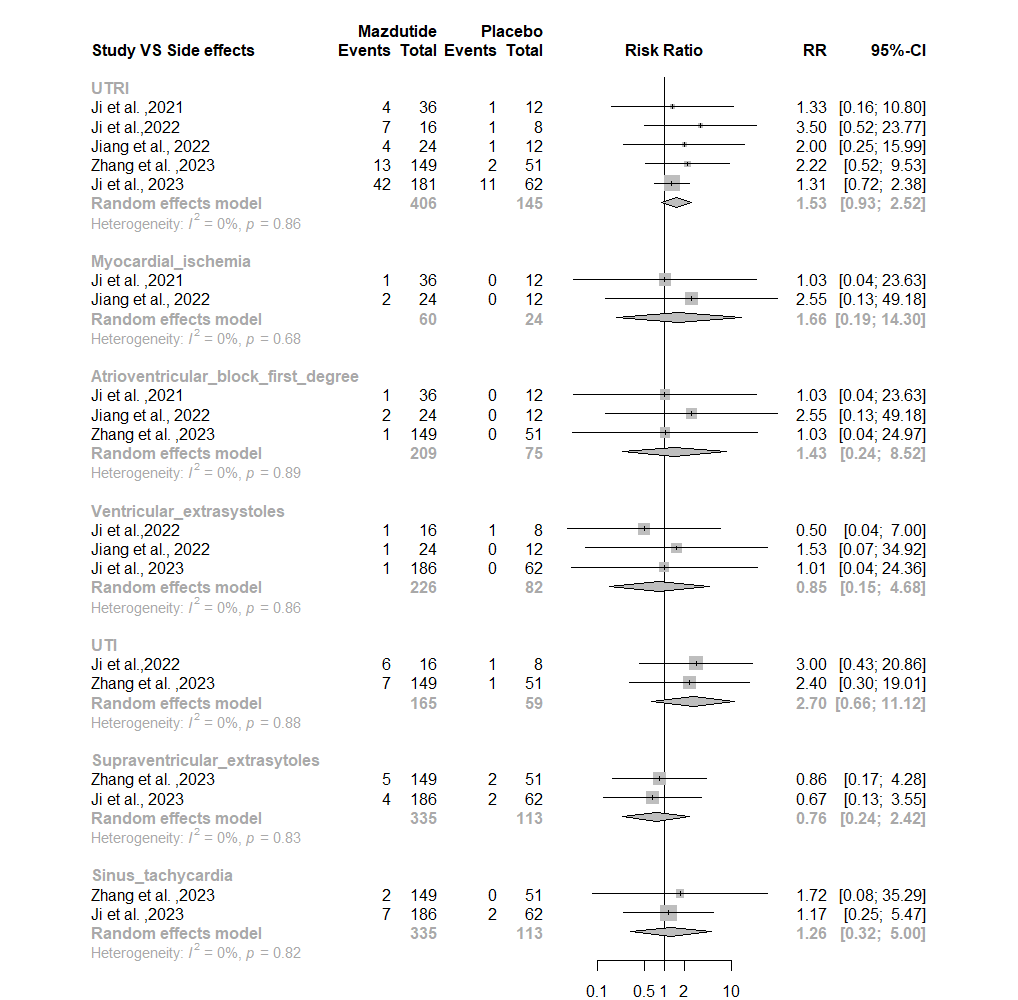

Supplement: Supplementary Figure 4 — Forest plot of the occurrence of non-gastrointestinal side effects of Mazdutide vs Placebo. [file Image_4.tiff]

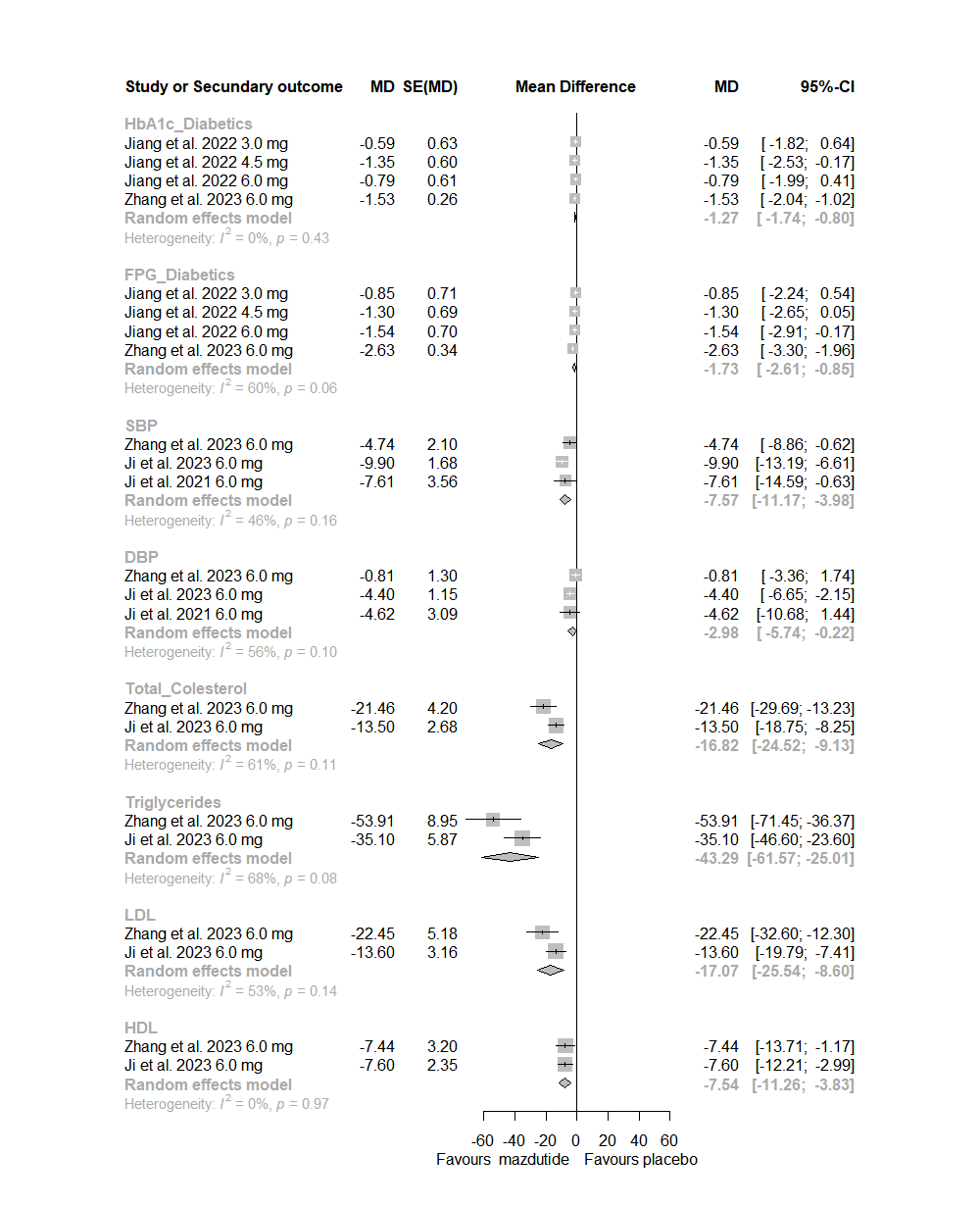

Supplement: Supplementary Figure 5 — Forest plot on the effect of Mazdutide vs Placebo on secondary outcomes. [file Image_5.tiff]
