# Supplementary material for: Efficacy and safety of Mazdutide on weight loss among diabetic and non-diabetic patients: a systematic review and meta-analysis of randomized controlled trials
Source: Front Endocrinol (Lausanne). 2024 Feb 14;15:1309118. doi: 10.3389/fendo.2024.1309118 (PMC10911117; doi:10.3389/fendo.2024.1309118)
Supplement: Supplementary file 6 [file Table_1.docx]

**Supplementary Table 1: Research query**

| **Database** | **Query** |
| --- | --- |
| Pubmed | #1: (“treatment efficacy” [Mesh] OR effectiveness OR Potency)  #2: Safe*  #3: (Mazdutide OR IBI362 OR LY3305677)     #4: (#1 OR # 2)      #5: #3 AND #4 |
| Web of Science | #1: ALL= (efficacy OR effectiveness OR Potency)     #2: ALL=(Safe*)     #3: ALL= (Mazdutide OR IBI362 OR LY3305677)     #4: #1 OR #2     #5: #3 AND #4 |
| Scopus | **#1: (efficacy OR effectiveness OR Potency) [All]**     #2:  Safe*  #3: (Mazdutide OR IBI362 OR LY3305677)     #4: (#1OR #2)     #5: #3 AND #4 |
| Cochrane library | ((efficacy OR effectiveness OR Potency) OR (Safe*)) AND (Mazdutide OR IBI362 OR LY3305677) |
| Google scholar | ((efficacy OR effectiveness OR Potency) OR (Safe*)) AND (Mazdutide OR IBI362 OR LY3305677) |
| Clinicaltrial.gov | ((efficacy OR effectiveness OR Potency) OR (Safe*)) AND (Mazdutide OR IBI362 OR LY3305677) |
